# MRI detects blood-brain barrier alterations in a rat model of Alzheimer’s disease and lung infection

**DOI:** 10.1038/s44303-025-00071-5

**Published:** 2025-03-04

**Authors:** Yolanda Ohene, William J. Morrey, Elizabeth Powell, Katherine F. Smethers, Nadim Luka, Kieron South, Michael Berks, Catherine B. Lawrence, Geoff. J. M. Parker, Laura M. Parkes, Hervé Boutin, Ben R. Dickie

**Affiliations:** 1 Division of Psychology, Communication and Human Neuroscience, School of Health Sciences, Faculty of Biology, Medicine and Health, University of Manchester, Manchester, UK; 2 Geoffrey Jefferson Brain Research Centre, Manchester Academic Health Science Centre, University of Manchester, Manchester, UK; 3 Department of Medical Physics and Biomedical Engineering and Department of Neuroinflammation, Centre for Medical Image Computing, UCL, London, UK; 4 Division of Neuroscience, School of Biological Sciences, Faculty of Biology, Medicine and Health, University of Manchester, Manchester, UK; 5 Division of Informatics, Imaging and Data Sciences, Faculty of Biology, Medicine and Health, University of Manchester, Manchester, UK; 6 Bioxydyn Limited, Manchester, UK; 7 Imaging Brain & Neuropsychiatry iBraiN, Université de Tours, INSERM, Tours, France

**Keywords:** Diseases, Imaging the immune system

## Abstract

Pneumonia is a common infection in people suffering with Alzheimer’s disease, leading to delirium, critical illness or severe neurological decline, which may be due to an amplified response of the blood-brain barrier (BBB) to peripheral insult. We assess the response of the BBB to repeated *Streptococcus pneumoniae* lung infection in rat model of Alzheimer’s disease (TgF344-AD), at 13- and 18-months old, using dynamic contrast-enhanced (DCE) MRI and filter exchange imaging. Higher BBB water exchange rate is initially detected in infected TgF344-AD rats. BBB water exchange rates correlated with hippocampus aquaporin-4 water channel expression in infected animals. We detected no differences in BBB permeability to gadolinium contrast agent measured by DCE-MRI, confirmed by staining for tight junction proteins, occludin and claudin-5. These findings provide insight into the mechanisms of how peripheral inflammation impacts the BBB.

## Introduction

Older people are more susceptible to contracting and suffering from common infections, such as urinary tract infection, SAR-CoV-2 virus or pneumonia, potentially leading to critical illness with neurological consequences^1–8^. In recent decades, the deleterious contribution of peripheral infection in the pathophysiology of dementia has been increasingly recognised^9^. Neuroinflammation has been implicated as a process that may link peripheral infection and neurological symptoms. Neuroinflammation is also now regarded as a key process that occurs in the prodromal stage of Alzheimer’s disease and other neurodegenerative disorders, which can be probed using PET ligands binding to activated microglia, and which correlates with and can predict cognitive decline^10–14^. Despite the progress achieved in understanding the role of neuroinflammatory and neurodegenerative processes associated with dementia, there remains a gap in the understanding of how peripheral infection interacts with the pathophysiology of Alzheimer’s disease. An emerging hypothesis is that infection has a more profound effect on patients with dementia because they exhibit pre-existing blood-brain barrier (BBB) dysfunction^15^, with primed glial cells having already triggered a pro-inflammatory signal cascade that makes the brain more vulnerable to peripheral inflammation^16^.

The BBB is an adaptive physical and biochemical barrier with the primary function of protecting the central nervous system. The BBB consists of endothelial cells with tight junction proteins, pericytes, microglia, and astrocytic endfeet. Alzheimer’s disease is characterised by the aggregation of amyloid-β and tau proteins in the brain. In recent years, it has emerged that BBB dysfunction occurs early in the pathogenesis of Alzheimer’s disease^17–19^, and importantly, correlates with cognitive decline^20–22^. Chronic or acute inflammation, as found in Alzheimer’s disease or infection respectively, can cause BBB dysfunction that disrupts the tightly regulated transfer and clearance of vital molecules and metabolic by-products, causing harm to the brain. In Alzheimer’s disease, this contributes to a decrease in amyloid-β clearance and subsequent increase in amyloid-β and tau aggregation^23^, as well as activation of microglia, astrocyte reactivity, and pericyte shedding^24^, which further destabilises the BBB. Studies in animal models of Alzheimer’s disease show that systemic inflammation induced by peripheral administration of lipopolysaccharide (LPS) can cause BBB damage and amyloidosis, increasing memory impairments^25–27^. Several studies demonstrate that LPS increases the expression of the perivascular aquaporin-4 (AQP4) water channel proteins located on astrocytic endfeet, increasing cytokine secretion and membrane permeability^28–30^. Recently, we showed that *Streptococcus pneumoniae* lung infection leads to an increase in water exchange across the BBB along with higher AQP4 expression in the rat brain, detected using non-invasive filter exchange imaging (FEXI) MRI^31^.

MRI is a powerful tool for measuring BBB alterations in dementia because it can be applied both in preclinical and clinical settings^32^. Dynamic contrast-enhanced (DCE) MRI has been used to measure early BBB disruption in Alzheimer’s disease by monitoring leakage of gadolinium-based contrast agents^19,32,33^, but requires careful implementation when probing subtle leakage^34–37^. Further advanced MRI techniques, using either contrast agent^38,39^ or endogenous blood water as a tracer^40,41^, are able to probe subtler non-disruptive changes, by measuring the rate of water exchange across BBB. BBB water exchange techniques have been successful in detecting BBB changes associated with ageing, vascular risk factors and Alzheimer’s disease both in animal models^42–44^ and humans^45–49^. An increase in BBB water permeability surface area product was measured in a rat model of Alzheimer’s disease (TgF344-AD), and a further study demonstrated Alzheimer’s-related pathology accelerated BBB changes as the animals aged relative to wild-types^43,44^. Importantly, BBB water exchange has been found to be significantly correlated with memory and cognition in patients with mild cognitive impairment (MCI)^45^. Zhang et al.^50^ also demonstrated that MCI and Alzheimer’s disease patients had higher BBB water exchange rates compared to age-matched controls, detected using the FEXI technique, and specifically poorer cognition was associated with increased water exchange in the hippocampus.

While these BBB techniques show promise in detecting subtler changes compared with DCE-MRI, the mechanisms driving the changes in BBB water exchange are still unclear. Furthermore, little is known about the comorbid impact of lung infection and Alzheimer’s disease on BBB^51^. Therefore, understanding the specific BBB alterations caused by peripheral inflammatory events in the Alzheimer’s disease brain, particularly those detectable by highly translatable MRI techniques, would be highly beneficial for identifying potential therapeutic targets that aim to ameliorate the effects of peripheral infection on the brain.

In this study, we aim to investigate the impact of lung infection on the BBB in the TgF344-AD rat model of Alzheimer’s disease. We do this by inducing *S. Pneumoniae* lung infection in TgF344-AD rats at 13-months and 18-months of age to assess the impact of repeated lung infection. We hypothesise that peripheral infection will exacerbate BBB dysfunction to a greater degree in transgenic rats and that the impact of infection worsens as the disease progresses. Non-invasive DCE-MRI and FEXI are used to track effects of infection on the BBB longitudinally over time. Immunofluorescence microscopy is then used to evaluate tight-junction proteins (occludin, claudin-5 and zona-occluden-1 (ZO-1)), AQP4 water channels and activated microglia to understand which BBB immune cell changes may be involved with peripheral and neuroinflammatory mechanisms in Alzheimer’s disease pathogenesis.

## Results

### No differences in blood-brain barrier permeability to gadolinium-based contrast agent in response to lung infection

**Fig. 1:**
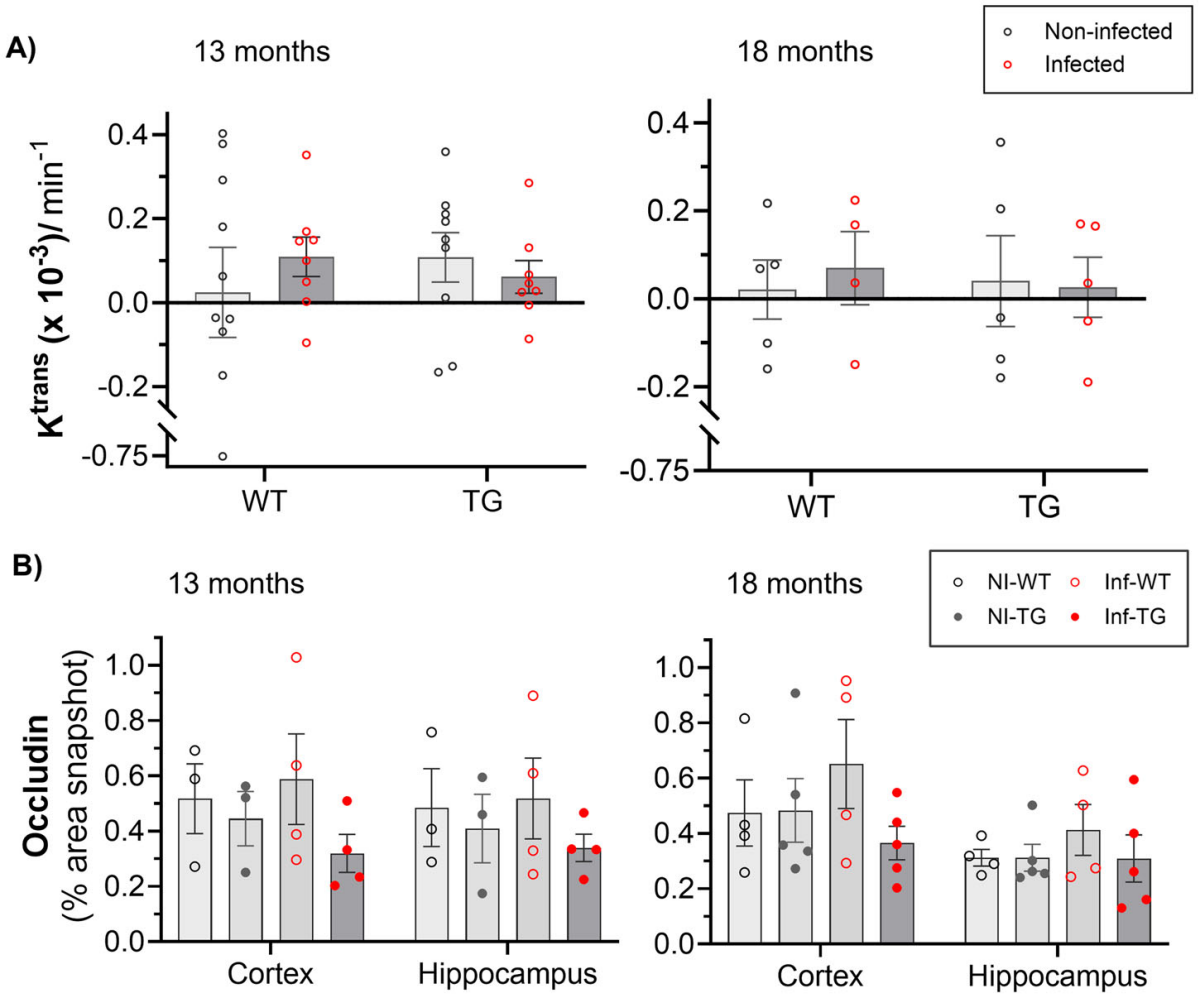
Blood-brain barrier volume transfer constant (*K*^trans^) and tight-junction protein in response to lung infection and Alzheimer’s disease. **A** *K*^*trans*^ in non-infected and infected wildtype (WT) and TgF344-AD (TG) animals at 13-month and 18-month timepoints. **B** Percentage area of the tight junction protein, occludin was measured in all groups, non-infected wildtype (NI-WT), infected wildtype (Inf-WT), non-infected TgF344-AD (NI-TG) and infected TgF344-AD (Inf-TG), at 13-months and 18-months timepoints. All plots indicate individual animal values and mean ± S.E.M.

DCE-MRI was used to assess paracellular BBB integrity via the volume transfer constant *K*^trans^. Low leakage rates were recorded across all animal groups. There was no measurable effect of genotype or infection on *K*^trans^, at either timepoint (Fig. 1A), as observed by inspecting the signal time courses and the model fits (Supplementary Fig. S1A). Table 1 provides *K*^trans^ values, group numbers and associated statistical analyses. At 13-months, the blood plasma volume (*v*_p_) was 13% higher in infected animals than in the non-infected animals (*P* = 0.04, ANOVA; Supplementary Fig. S1B, Supplementary Table S1). This difference was not replicated at the 18-month reinfection timepoint, where there were no significant effects of genotype or infection on *v*_p_. At 13-months, the whole brain *T*_1_ relaxation time was 1.5% higher in infected animals compared to non-infected animals (*P* = 0.009; ANOVA), with the greatest difference measured in TG animals (+1.8%; adjusted *P* = 0.04, post-hoc multiple comparisons test; Supplementary Fig. S1C, Supplementary Table S1). At the 18-month timepoint, the effect of infection on *T*_1_ was no longer present (Supplementary Fig. S1C).

**Table 1:** Imaging parameters with statistical analysis

Imaging Parameter	Values (Mean ± S.E.M)				Two-way ANOVA				
	**Wild-type**		**TgF344-AD**					Multiple comparisons	
	Non-Infected	Infected	Non – infected	Infected		F-value	P-value		Adjusted P-value
*K*^trans^ (x10^-4 ^min^-1^)	**13 months**								
	*(n* = 10*)* 0.24 ± 1.07	*(n* = 8*)* 1.09 ± 0.47	*(n* = 9*)* 1.08 ± 0.59	*(n* = 8*)* 0.61 ± 0.39	Infection	0.07	0.79		
					Genotype	0.06	0.81		
					Interaction	0.78	0.38		
	**18 months**								
	*(n* = 5*)* 0.21 ± 0.66	*(n* = 4*)* 0.70 ± 0.83	*(n* = 5*)* 0.40 ± 1.03	*(n* = 5*)* 0.26 ± 0.68	Infection	0.04	0.84		
					Genotype	0.02	0.89		
					Interaction	0.15	0.15		
*k*_in_ (s^-1^)	**13 months**								
	*(n* = 16*)* 2.82 ± 0.26	*(n* = 15*)* 3.56 ± 0.50	*(n* = 10*)* 2.17 ± 0.40	*(n* = 10*)* 5.47 ± 1.29	Infection	9.38	**0.004	Non-infected – Infected	
					Genotype	0.87	0.35	WT	0.37
					Interaction	3.66	0.06	TG	*0.005
	**18 months**								
	*(n* = 6*)* 3.38 ± 0.55	*(n* = 4*)* 3.28 ± 0.60	*(n* = 7*)* 2.50 ± 0.52	*(n* = 7*)* 3.24 ± 0.41	Infection	0.36	0.55		
					Genotype	0.76	0.39		
					Interaction	0.63	0.63		

*WT* wildtype, *TG* TgF344-AD.

Gadolinium-contrast agents leak through tight junction gaps; concordant with the DCE-MRI data, we found no measurable differences in occludin or claudin-5 area between any of the groups at either of the timepoints, suggesting no gross disruption to the BBB (Fig. 1C, Supplementary Fig. S2A respectively). ZO-1 was significantly lower in transgenic group than in wildtype animals in the cortical region at 13-months, which would suggest a weakening of the tight junction anchoring protein (Supplementary Fig. S2B). At 18-months, this difference in ZO-1 was no longer present.

### Increased blood-brain barrier permeability to water in TgF344-AD rats in response to lung infection

**Fig. 2:**
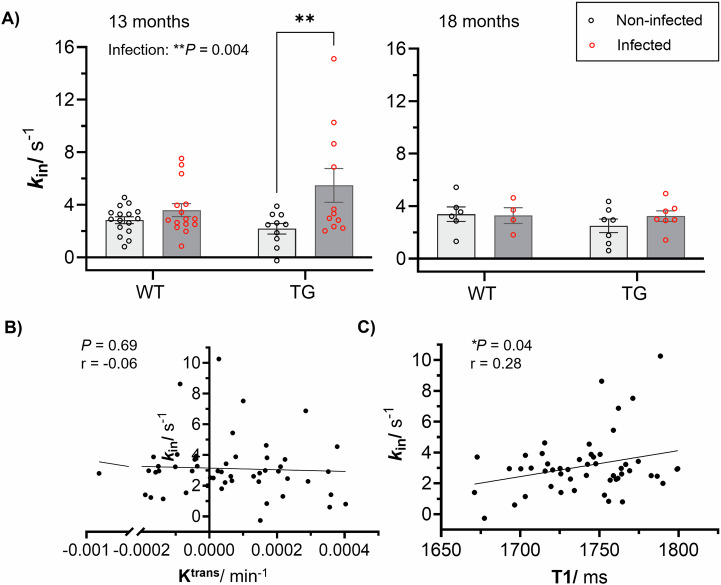
Blood-brain barrier water exchange rate (*k*_in_) and association with *K*^trans^ and *T*_1_. **A** Individual *k*_in_ measurements for non-infected and infected wildtype (WT) and TgF344-AD (TG) animals at 13-months old (**adjusted *P* = 0.005; post-hoc) and 18-months old. Individual animal values and mean ± S.E.M, and significant ANOVA results displayed on the plots. **B** *K*^trans^ against *k*_in_ for all animals at both timepoints (*P* = 0.69; *r* = −0.06; *n* = 51). **C** T1 association with *k*_in_ for all animals at both timepoints (*P = 0.04; *r* = 0.28; *n* = 51).

To assess subtle and, potentially, non-disruptive alterations to the BBB, we applied non-invasive FEXI MRI to measure the BBB water exchange rate (*k*_in_) (Fig. 2A). At 13-months old, ANOVA main effect analyses detected a significant effect of infection on *k*_in_ (*P* = 0.004). In post-hoc analysis, the infected TG group showed 152% higher *k*_in_ than the non-infected TG animals (adjusted *P* = 0.005; post hoc multiple comparisons). At 18-months, no effect of infection or genotype was observed. Table 1 provides *k*_in_ values, group numbers, and corresponding statistical analyses. Mean normalised BBB water exchange rate recovery curves are presented in Supplementary Fig. S3A. At 13-months, there was a significantly higher extravascular diffusivity (*D*_e_) due to genotype (*P* = 0.0007; ANOVA), specifically in infected TG group (adjusted *P* = 0.0098; post hoc multiple comparisons; Supplementary Fig. S3B). We detected no differences due to infection or genotype at either timepoint in the intravascular diffusivity (*D*_i_) (Supplementary Fig. S3C). In a paired analysis of non-infected and infected WT and TG animals no significant differences were measured due to either age or genotype (Supplementary Fig. S5A).

No correlation was found between *K*^trans^ and *k*_in_, which suggests that the two imaging metrics capture different BBB properties (Fig. 2B). There was an association between *T*_1_ and *k*_in_ measurements, which may suggest a shift in the water balance due to BBB alterations (Fig. 2C).

### Lung infection upregulates aquaporin-4 water channel protein

**Fig. 3:**
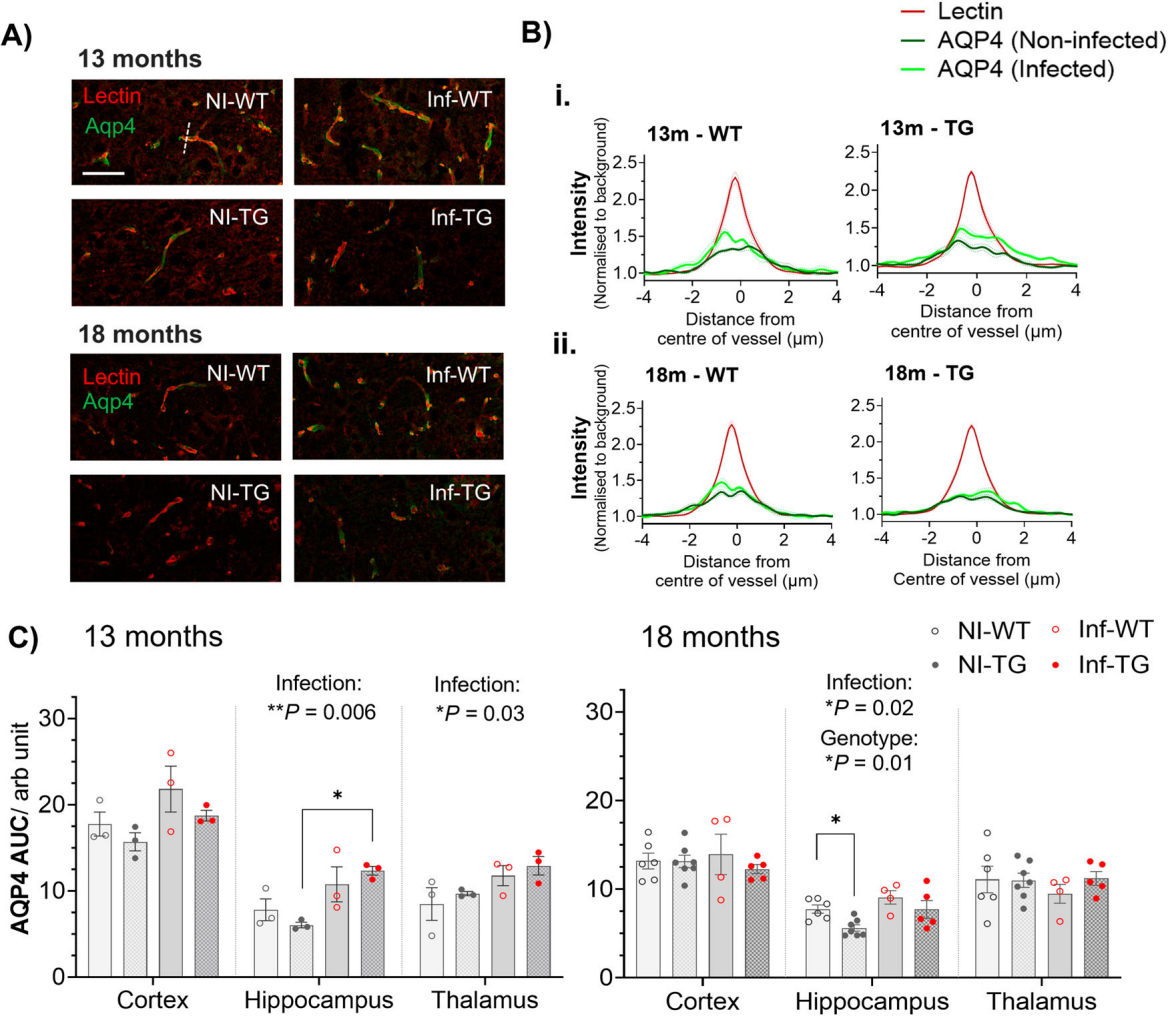
Aquaporin-4 water channel protein quantification. **A** Representative immunofluorescence images taken in the cortex of non-infected wildtype (NI-WT), non-infected TgF344-AD (NI-TG), infected wildtype (Inf-WT) and infected TgF344-AD (Inf-TG) animals at each timepoint. The solid white line is a scale bar of 50 μm and the white dashed line is an example line profile. **B** Mean intensity profiles taken from the hippocampal brain region of lectin (red) and AQP4 (dark green - non-infected; light green – infected) *at* **i***.* 13-months (all groups, *n* = 3) and **ii**. 18-months old (NI-WT, *n* = 6; Inf-WT, *n* = 4; NI-TG, *n* = 7; Inf-TG, *n* = 5). **C** Individual area under curve (AUC) of AQP4 in the cortex, hippocampus and thalamus at 13-months (*adjusted *P* = 0.01; post-hoc) and at 18-months (*P = 0.04). Individual animal values are presented with mean value ± S.E.M, and significant ANOVA results indicated on each plot.

Following the observation of increased BBB water-exchange in infected rats, we sought to determine the impact of infection on the AQP4 protein, a water-channel protein located on astrocyte endfeet and closely interfaced with the microvasculature. As such, we measured the area under the curve (AUC) of AQP4 staining intensity across the capillaries (Fig. 3A).

The mean lectin and AQP4 intensity profiles for the hippocampus showed an upregulation of AQP4 following initial infection at 13-months old (Figure 3Bi), which was no longer present at 18-months old (Fig. 3Bii). AQP4 intensity profiles for the cortex and thalamus brain regions are presented in Supplementary Fig. S4. ANOVA main effect analysis showed that infection had a significant effect on the AQP4 AUC at the 13-month timepoint for hippocampus (*P* = 0.006) and thalamus (*P* = 0.03), and a non-significant trend towards an increase in the cortex (*P* = 0.06), Fig. 3C. In post-hoc testing, the hippocampus presented a markedly higher AQP4 AUC in the infected TgF344-AD animals than in their non-infected counterparts (+107%, adjusted *P* = 0.01). When animals were reinfected at 18-months, the hippocampus ANOVA showed significant upregulation in AQP4 due to infection (+25%, *P* = 0.02) and significantly lower AQP4 in the TgF344 rats than in their WT counterparts (−21%, *P* = 0.01). The largest difference in AQP4 was measured between non-infected wildtype and non-infected TgF344 animals (−28%, adjusted *P* = 0.04; post-hoc multiple comparison). No measurable differences in any groups were found in the cortex or thalamus regions at 18-months (Fig. 3C). Quantitative values, group numbers and statistical analyses can be found in Supplementary Materials, Supplementary Table S2.

### Lung infection activates microglia in WT rats, while both non-infected and infected TgF344-AD show activation

**Fig. 4:**
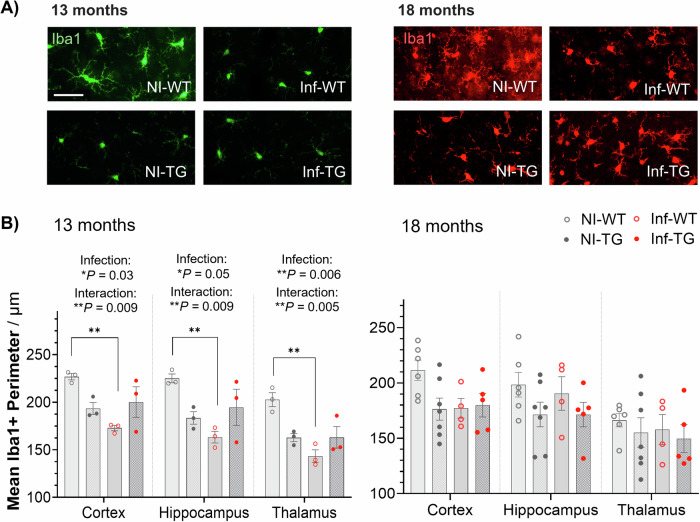
Microglia response to lung infection and Alzheimer’s disease gene. **A** Representative Iba1+ immunofluorescence images for microglia in the cortex of non-infected and infected wildtypes (NI-WT/ Inf-WT) and non-infected and infected TgF344-AD rats (NI-TG/Inf-TG) at 13-months (green) and 18-months (red). The white line is a scale bar of 50 μm. **B** Mean Iba1+ perimeter across individual animals in three brain regions, cortex (adjusted **P = 0.005), hippocampus (adjusted **P = 0.007) and thalamus (adjusted **P = 0.001) at 13-months and 18-months. Individual animal values are presented with mean value ± S.E.M, and significant ANOVA results indicated on each plot.

Microglial activation was determined by measuring the mean perimeter of Iba1 positive cells. Lower values indicate an increase in activation as the microglia transform towards an ameboid morphology (Fig. 4A). When initially infected at the 13-month timepoint, 2-way ANOVA found that the Iba1+ perimeter was significantly lower due to infection in all brain regions (cortex, −12%, *P* = 0.03; hippocampus, −12%, *P* = 0.05; thalamus, −16%, *P* = 0.006). Following post-hoc multiple comparisons, the mean perimeter of Iba1+ cells was significantly lower in infected WT animals than in non-infected WT in the cortex (−25%, adjusted *P* = 0.005; post-hoc), hippocampus (−28%, adjusted *P* = 0.004; post hoc) and thalamus (−31%, adjusted *P* = 0.001; post hoc). At 18-months, no measurable differences were found in any of the groups in any brain region. When assessing differences between 13-months and 18-months old rats in the hippocampus, mean microglia perimeter in the non-infected group was significantly lower in TgF344-AD rats than wildtype rats (−16%, *P* = 0.01; ANOVA) (Supplementary Fig. S5C). No measurable difference due to age or transgenic status was found in the infected group. All quantitative values and statistical analyses can be found in Supplementary Materials, Table S3.

### Hippocampal aquaporin-4 is associated with increased blood-brain barrier water exchange and microglial activation

**Fig. 5:**
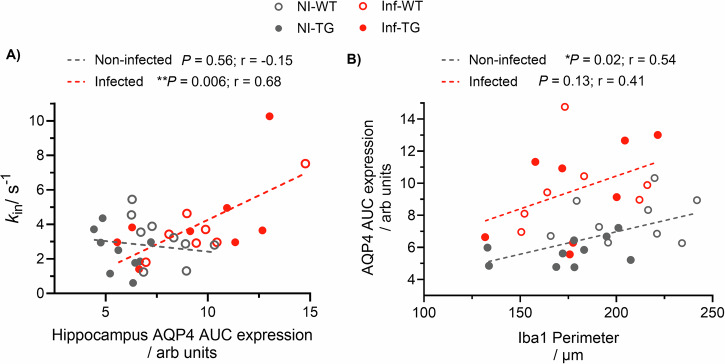
Association of aquaporin-4 (AQP4) with BBB water-exchange rate (*k*_in_) and microglia (Iba1 + ) perimeter across all animals. **A** The association between the hippocampus AQP4 AUC and *k*_in_ for non-infected animals (*P* = 0.56; *r* = −0.15; *n* = 18, black dashed line) and infected animals (**P = 0.006; *r* = 0.68; *n* = 15, red dashed line). **B** Mean hippocampus Iba1 perimeter against hippocampus AQP4 AUC for the non-infected group (*P = 0.02; *r* = 0.54; *n* = 19, black dashed line) and infected group (*P* = 0.13; *r* = 0.41; *n* = 15, red dash line).

Since the hippocampus is a key brain region affected by Alzheimer’s disease this region was further investigated. There was a significant positive correlation between AQP4 expression in the hippocampus and *k*_in_ in infected animals (*P* = 0.006), demonstrating the potential sensitivity of the non-invasive water exchange measurements to perivascular AQP4 following peripheral insult (Fig. 5A).

Significant changes were also found in the hippocampus region for AQP4 water channel protein due to both infection and genotype, the association between AQP4 expression and Iba1+ perimeter was further explored for both non-infected and infected groups in this brain region (Fig. 5B). In the non-infected group (NI-WT and NI-TG), a significant positive correlation was found between microglial activation in the hippocampus and AQP4 expression (*P* = 0.02), which indicates a possible interaction between perivascular AQP4 and microglia. The association was weaker and non-significant within the infected group (*P* = 0.13) (Fig. 5B).

## Discussion

Pneumonia is a common infection in individuals suffering from Alzheimer’s disease, which can lead to accelerated cognitive decline^7^. In this study, we use advanced MRI techniques and immunofluorescence to assess the response of the BBB to repeated *Streptococcus pneumoniae* lung infection, in a rat model of Alzheimer’s disease (TgF344-AD). We find that at 13-months infected rats had significantly higher BBB water exchange rate than their non-infected counterparts, mainly driven by a 152% higher *k*_in_ in infected compared to non-infected TgF344-AD rats. Our results suggest that there is an upregulation of AQP4 proteins during infection, which correlated with BBB water exchange measurements, particularly in the hippocampus region, which is affected early in the pathogenesis of Alzheimer’s disease^19,50^. An association between microglia and AQP4 is also exhibited in the hippocampus of the non-infected animals, via a positive correlation between Iba1+ perimeter and extent of AQP4 protein surrounding the microvasculature. We detected no differences in BBB permeability to gadolinium contrast agent, as measured by conventional DCE-MRI (via *K*^trans^), probably due to the lack of disruption to tight junction proteins. Occludin and claudin-5 were maintained across the duration of the experiment. This suggests that our model of mild lung infection does not cause gross physical disruption to the BBB.

Peripheral infection is known to have an impact on the BBB, particularly in conjunction with Alzheimer’s disease pathology^16^. To confirm animals were infected, *S. pneumoniae* bacteria and antibodies were identified using appropriate antibodies, and both were observed in the lung tissue of infected animals (Supporting Materials, Supplementary Fig. S6). To our knowledge, there have been no imaging studies assessing the impact of lung infection on the BBB in the Alzheimer’s disease brain. Takeda et al. previously reported exacerbated BBB vulnerability in a mouse model of Alzheimer’s disease (expressing human amyloid precursor protein (APP)), and behavioural changes worsened when animals were given a peripheral LPS challenge^26^. Increased BBB leakage in the brain tissue was measured via the permeability of fluorescent albumin protein, suggesting gross BBB disruption due to the large size of this molecule (67 kDa). Our data indicate that *S. pneumoniae* lung infection induces a comparatively mild peripheral inflammatory stimulus relative to LPS^52^, with no gross behavioural changes or sickness behaviour noted during the infection period. This is in agreement with a previous study that reported that *S. pneumoniae* did not affect the motor and memory performance or amyloid-β deposition in transgenic (Tg25676) Alzheimer’s disease mice^51^. As well as changes to *k*_in_, we noted a significant increase in *T*_1_ relaxation time due to infection at 13-months (Supplementary Fig. S1C). While *k*_in_ reflects the forward-rate constant of bi-directional exchange, it is possible that disruption to BBB water handling may cause a shift in brain water balance.

Our result supports results from several studies suggesting that changes in AQP4 occur in pathological conditions^53,54^ i.e., with induced meningitis^55,56^, ischemic stroke^57^ or in hypoxic conditions^29^. Song et al.^29^ demonstrated that pro-inflammatory factors such as LPS, TNF-α and IL-1β increase AQP4 protein and mRNA expression, and correlate with membrane permeability. Since groups size used for AQP4 analysis were small for the first timepoint (*n* = 3), a larger cohort should be used in future work to confirm our finding. Alternative MRI water exchange measurement techniques (e.g., multiple echo-time ASL and ^17^O-water-MRI) have previously reported sensitivity to AQP4 using AQP4^-/-^ knockout mice, which provides further evidence to support the sensitivity of water exchange MRI to the AQP4 protein^58,59^. Future studies that transiently alter the function of AQP4 could further probe the relationship between the water channels and the non-invasive MRI measures, and their interaction with other components of the BBB.

Our data show that in wildtype rats *S. pneumoniae* activates the resident immune cells within the brain, as measured by a lower Iba1+ perimeter. In TgF344-AD rats, Iba1+ perimeter was already reduced, suggesting that microglia were already in an activated or primed state. Infection did not modulate Iba1+ perimeter further (Fig. 4B)^60–62^. In non-infected animals, hippocampal microglial perimeter values positively correlated with lower hippocampus perivascular AQP4. The association seems to be dampened in the infected group where both microglia activation and AQP4 upregulation occurs (Fig. 5B).

We found minimal response to the infection challenge when animals were reinfected at 18-months old. It is possible this was due to a build-up of antibodies against *S. pneuomoniae*, which enabled a faster inflammatory response when animals were reinfected. Previous reports demonstrate that the response of the pro-inflammatory marker IL-1β is lower when a low-grade LPS challenge is repeated in a *App*^NL-G-F^ mouse model of Alzheimer’s disease, which suggest a dampening of the immune response of animals in the laboratory setting after multiple inflammatory insults^63^. No differences in BBB water exchange rate due to age were measured in this study (Supplementary Fig. S5A); more substantial changes due to aging have previously been reported and emphasise the deterioration of BBB integrity with age^42,43,47,49^. The group numbers were lower than expected at the 18-months timepoint as six rats had to be culled for unrelated health reasons. It is important that our (neutral) findings regarding the impact of repeated infection and Alzheimer’s disease on BBB water exchange is replicated in a larger cohort of older rats.

In this study, we measure the BBB forward water exchange rate (*k*_in_) using a crusher gradient compensated model, which minimises the impact of cross-term effects on *k*_in_ arising due to use of crusher gradients with variable mixing times^31,64^. It has been reported that FEXI may be affected by changes to tissue microstructure^65^. However, our application of FEXI probes exchange between intravascular and extravascular compartments, and is likely to be far less sensitive to microstructural alterations in tissue compared to applications of FEXI that use higher filter b-values to probe transcytolemmal exchange. Future studies modulating filter b-value would provide further insight into the intravascular and extravascular contributions nulled using very low b-values.

There were no effects of genotype or infection on DCE-MRI-derived *K*^trans^ at either timepoints during the disease progression. This result corroborates previous findings in the TgF344-AD rat model at 13-months and 18-months old^43,44^. Here, we find that this level of infection does not induce disruptive alterations at the BBB, which arise with tight junction breakdown, since no difference in occludin or claudin-5 was measured. Our measurements show no correlation between *k*_in_ and *K*^trans^, which would suggest that the two MRI techniques may probe different BBB pathways. Comparison between water exchange and contrast agent permeability has been previously reported in the human brain in an elderly population using DCE-MRI and diffusion prepared-ASL techniques; this study found no association between the two techniques across whole brain regions^66^.

A limitation of this study is that robust regional estimates of BBB water permeability were not possible using our FEXI protocol. The frontal cortex and the hippocampus would be key brain regions to target for BBB water exchange measures in the context of neuroinflammation and Alzheimer’s disease. Technical improvements to the FEXI technique, such as incorporating phase cycling, could be implemented in future studies to attempt to increase the SNR for robust regional measurements^67^. This could enable the assessment of smaller brain regions, which has not yet been successfully established using rodent FEXI-MRI protocols. Genotype differences in BBB water permeability surface product (*PS*_w_) in the TgF344-AD rat model, especially in the hippocampus, were reported at 18-months old in previously studies using the MFAME-MRI technique^44^. We did not replicate this finding using our FEXI protocol, possibly because of dilution effects caused by using a whole brain ROI. MFAME-MRI could be considered as alternative to FEXI for rodent studies for regional estimates, though it requires high doses of contrast agent which is not possible to use in a clinical setting. FEXI has been used to measure BBB water exchange in the hippocampus of Alzheimer’s disease patients, therefore a clinical study assessing patients who also have an infection would be feasible. Finally, using both male and female rats were beyond the scope of this study, but female rats should be assessed in the future to determine any sex differences in BBB function^68^.

This study characterises the impact of repeated lung infection on BBB alterations in TgF344-AD animals at different stages of the Alzheimer’s disease progression. We demonstrate the sensitivity of FEXI MRI as a clinically translatable tool in detecting non-disruptive BBB alterations due to infection, emphasising the utility of MRI in capturing subtle BBB dysfunction in the Alzheimer’s disease brain. Aquaporin-4 water channels and microglia are found as key BBB components in responding to peripheral infection, however their role (protective or deleterious) is yet to be determined.

## Methods

### Animals

Male TgF344-AD (TG) rats, exhibiting progressive accumulation of amyloid and tau pathologies to mimic human disease^62,69^, and their wild-type (WT) littermates were used in this study. Sample sizes were calculated to detect an effect of infection on MRI measures of BBB permeability from previous data^53^ see Power Calculation. Animals were randomised into four groups: non-infected WT (NI-WT; *n* = 16), non-infected TG (NI-TG; *n* = 11), infected WT (Inf-WT; *n* = 15), and infected TG (Inf-TG; *n* = 11). Experimental procedures were approved by the Preclinical Imaging Executive Committee of the University of Manchester and carried out in accordance with the UK Animals (Scientific Procedures) Act 1986 and EU Directive 2010/63/EU for animal experiments. Breeding, housing and husbandry details conformed to the ARRIVE guidelines^70^.

Two male and two female wild-type (WT) Fischer and TgF344-AD rats with the APPswe and PS1Δe9 mutations were purchased from the laboratory of Prof T. Town (University of Southern California) and were set up as breeding pairs, housed in the Biological Services Unit at the University of Manchester. Genotyping was outsourced to TransnetyxR, with a total of 26 WT and 22 TgF344-AD animals used in the present study. All animals were housed in groups of 2–4 per cage with individual ventilation, environmental enrichment, *ad libitum* access to food and water and a 12:12 h cycle of light and dark for the whole duration of the study. Animals in the infected group were housed in quarantine block for the duration of the study to avoid infection contamination or spread.

### Streptococcus pneumoniae lung infection protocol

A protocol for doses and timing of *Streptococcus pneumoniae* (American Type Culture Collection [ATCC] 49619, capsular serotype 19 F [Danish]) challenge was established to allow low-grade, indolent pulmonary exposure to *S. pneumoniae* infection that is sustained in animals for a period of 7–8 days^52^.

*S. pneumoniae* bacteria were plated on sheep blood agar (Becton, Dickinson and Company) and incubated for 24 h at 37 °C with 5% CO_2_. 15–20 colonies were added to 25 ml of growth media (brain heart infusion (Becton, Dickinson and Company) supplemented with 20% of heat-inactivated foetal bovine serum (HIFBS)) which was incubated on an agitator at 37 °C in 5% CO_2_ atmosphere until an optical density, measured using a spectrophotometer, of 0.6 (mid-log phase) was reached. The concentration was calculated using the Miles Misra technique^71^. These cultures were centrifuged at 9 G and pellets were stored at −80 °C as stock inoculates. Stock was washed in sterile PBS, re-suspended and 50 μl was delivered intranasally between the two nostrils in an ascending challenge at concentrations of 4 × 10^8^ cfu/ml (day 0), 8 × 10^8^ cfu/ml (day 2) and 16 × 10^8^ cfu/ml (day 5). Weights were monitored during the infection protocol to ensure experimental endpoints were not reached (Supplementary Fig. S7). Animals were imaged on day 7/8, and following recovery from MRI, infected rats were administered with an oral dose (40 mg/kg) of amoxicillin antibiotic on day 8/9. Infected animals were reinfected using the same infection protocol six months later. None of the infected rats showed any signs of neurological symptoms during the infection challenge.

Each animal group was imaged using DCE-MRI and FEXI MRI on day 7–8 of the infection challenge (aged 12.7 ± 1.1 months, weight 384–539 g and aged 18.0 ± 0.2 months, weight 416–534 g). Brain and lung tissue was taken for immunofluorescence from a sub-group of animals (*n* = 3 per group) at 13-months old and the remaining tissue taken at 18-months old. Six animals were included in the NI-WT and Inf-WT group from a previous study in wild-types, these animals were scanned with FEXI-MRI before and after infection^31^ but not DCE-MRI. Six animals (NI-WT: *n* = 2; NI-TG: *n* = 2; Inf-WT: *n* = 2) were humanely culled between the two experimental timepoints due to unrelated health issues.

### MRI

For MRI scans, the animals were induced with 4% isoflurane anaesthesia and maintained under 2% isoflurane mixed into O_2_ at 0.7 L/min. Animals were cannulated in the tail vein with a 24 G catheter (Jelco) which was flushed with 0.1 ml saline before the scan. Animals were secured into the MRI cradle with a nose cone, ear bars and bite bar to minimise head movements. Core body temperature was monitored using a rectal probe (SA Instruments) and maintained at 36.5 ± 0.5 °C using a feedback-controlled hot air blower. A line containing Gd-DOTA (Dotarem, Guerbet) was attached to the cannular and connected to a syringe driver ready for injection during the scan.

Imaging data was acquired on a Bruker Avance III console (max gradient strength = 375 mT/m; max slew rate = 3375 T/m/s) interfaced with an Agilent 7 T 16-cm bore magnet. Radiofrequency transmission was performed by a Bruker transmit-only resonator (T11070V3) and a two-channel Bruker rat brain surface coil (T11205V3) was used for signal reception. An anatomical reference scan was acquired using a T2-TurboRARE sequence for positioning of DCE and FEXI imaging slices. This was followed by FEXI then DCE-MRI data collection with a total scan duration of 58 min 28 s for each animal.

### Dynamic contrast-enhanced MRI

All DCE-MRI quantities, models and processes described conform to the standards set out by OSIPI CAPLEX (v1.0.3)^72^. For full clarity, quantities, processes, and model references can be found with their CAPLEX definition here: (https://osipi.github.io/OSIPI_CAPLEX/).

DCE-MRI was used to measure the BBB volume transfer constant of gadolinium contrast agent, *K*^trans^ (min^-1^), the relative volume fraction of blood plasma, *v*_p_ (mL/mL) and native longitudinal relaxation rate *R*_1,0_ (s^-1^). For the DCE-MRI acquisition, *R*_1,0_ was estimated using variable flip angle (VFA) 3D spoiled gradient echo (SPGR) scans with acquisition parameters: prescribed excitatory flip angle = 2°, 5°, 12° and 20°, repetition time/ echo time (TR/TE) = 6.4/2.0 ms, voxel size = 0.27 × 0.27 × 1.0 mm^3^, matrix size: 128 × 128 × 20 and 4 signal averages. Dynamic 3D SPGR images were acquired with a prescribed excitatory flip angle = 20° before and during intravenous injection (i.v.) of contrast agent, Gd-DOTA (Dotarem, Guerbet) with 0.1 mmol kg^−1^ dose delivered though a 24 G catheter by an electronic syringe driver at 1.0 mL min^−1^. The acquisition parameters for these volumes were: TR/TE = 6.4/2.0 ms, voxel size = 0.27 × 0.27 × 1.0 mm^3^, matrix size: 128 × 128 × 20 and repetitions = 68; DCE scan duration = 18 min 28 s. The intravenous injection of contrast agent was unsuccessful in eight animals at 13-months and in four animals in the 18-month group.

The DCE-MRI data were analysed using the Madym software v4.21.2^73^ in Matlab R2021a (Mathworks). All images for each subject were co-registered to the first image using rigid registration in ELASTIX (version 5.0.1). Regions of interest (ROIs) were manually segmented across four slices that matched the location of the single thicker FEXI slice as detailed in the section below. The mean native longitudinal relaxation rate within the ROI was computed and used to calculate the mean change in longitudinal relaxation rate from the dynamic signal intensities. The timeseries of longitudinal relaxation rates was converted to indicator concentrations, *C*_t_ (mM) using the longitudinal relaxivity *r*_1_ = 3.2 s^−1 ^mM^-1^^74^. An arterial input function was selected by manually drawing a small ROI (~5 voxels) across the sagittal sinus region where the artery bifurcates in the brain. Contrast agent concentrations in arterial blood *C*_a,b_ were calculated from the AIF signal using the same method as described above. Whole blood concentrations were converted to concentrations in arterial plasma, *C*_a,p,_ assuming a haematocrit of 0.42. The Patlak model was then used to determine *K*^trans^ and *v*_p_ from *C*_a,p_ and *C*_t_, with limits to −0.001–0.1 min^-1^ for *K*^trans^ and 0.0–1.0 for *v*_p_ to constrain the fitting.

### Filter exchange imaging

FEXI was used to assess the BBB water exchange rate (*k*_in_) as described previously^31^. Briefly, FEXI is a double diffusion-encoding sequence that was originally developed to measure water exchange across cell membranes^75,76^, with recent application to estimate BBB water exchange^31,77,78^. Full details of the FEXI sequence and the *k*_in_ model which compensates for the crusher gradients can be found in ref. ^31^.

The parameters used in the FEXI protocol were: filter b-value *b*_*f*_ = 250 s/mm^2^ with gradient separation *∆*_f_ = 10 ms, gradient duration *δ*_f_ = 4 ms and filter echo time TE_f_ = 16.5 ms, mixing times *t*_m_ = 0.025, 0.05, 0.1, 0.2 and 0.3 s and detection b-values *b* = 0, 250 s/mm^2^ with diffusion gradient separation ∆ = 10 ms and duration δ = 4 ms, and TE = 35.5 ms. Images were acquired using a spin-echo EPI readout with readout direction = left-right and TR = 5000 ms. The acquisition consisted of a single slice = 4 mm thickness, with matrix size = 64 × 64 and FOV = 32 × 32 mm^2^ to give voxel resolution = 0.5 × 0.5 × 4.0 mm^3^ and 10 repetitions. An additional dataset with the filter block switched off (*b*_f_ = 0 s/mm^2^) was acquired at the shortest mixing time (*t*_m_ = 0.025 s), with all other imaging parameters the same as above, to provide an estimate of ADC. All diffusion gradients were applied in three orthogonal directions (XY, YZ, and XZ) for both filter and detection blocks. FEXI scan duration was 35 min.

Analysis of the FEXI data was performed in Matlab R2021a (Mathworks). All images for individual animals were registered to the first b0 image using rigid registration in ELASTIX (version 5.0.1). The geometric mean of the signal was computed from three orthogonal directions and repetitions for both filtered and non-filtered data and the resulting signal used to calculate ADC’ and ADC maps respectively, at each *t*_m_. Regions of interest covering the whole brain were manually drawn on the ADC maps, and a threshold applied to pixels with diffusivity in the range 0.65 to 1.0 × 10^−3^ mm^2^/s to eliminate voxels contaminated with CSF. The resulting ROI was used to extract an ROI-averaged ADC’ and ADC value for each *t*_m_.

Individual *k*_in_ values and intravascular diffusivity (*D*_i_) were calculated, with extravascular diffusivity (*D*_e_) fixed to ADC’(*t*_m_ = 0.025 s) for each animal and intravascular volume fraction (*f*_i_) was fixed at 0.02 as previously measured^31^. The limits of *k*_in_ were set between −1.0 and 20.0 s^−1^ to constrain the fitting. Two animals (one NI-WT and one NI-TG) were excluded as outliers from the 13-month FEXI dataset, detected using the ROUT method outlier test with false discovery rate set to Q = 1%^79^.

### Immunofluorescence

The BBB tight junction proteins: occludin, claudin-5 and zona-occluden-1 (ZO-1), as well as the aquaporin-4 water channel protein (AQP4) and microglia were assessed ex-vivo using immunofluorescence to investigate potential alterations to the BBB structure in response to lung infection and genotype. Details of antibodies used during immunofluorescence experiments are given in Supplementary Materials, Supplementary Table S4.

Following trans-cardiac perfusion with heparinised saline under isoflurane anaesthesia (5% induction and maintenance), excised brain hemispheres were immerse-fixed in 4% PFA for 24 h before cryoprotection in 20% sucrose before being snap frozen. Hemispheres were cut sagittally into 20 μm sections using a cryostat (Leica CM3050S). The sections were fixed for 10 min in 4% paraformaldehyde (PFA) in PBS. Following six washes in PBS, sections were incubated in 2% non-animal blocker permeabilizing-blocking solution (Vector Laboratories, SP-5030 and 0.1% triton X-100 (Sigma-Alrich, T9284) in PBS for 30 min, then incubated overnight at 4 °C in primary antibody (Supplementary Table S4) in permeabilizing-blocking solution. Sections were then incubated in a secondary antibody (Supplementary Table S4) in permeabilizing-blocking solution at room temperature for 2 h. Sections were washed again in PBS before being mounted with prolong gold antifade kit (Thermo-Fisher, P36934).

All images were acquired at 20× magnification on a fluorescence microscope (Olympus U-RFL-T) using QCapturePro7 software. Images were captured in the posterior cingulate/temporal cortex, hippocampus and thalamus, matching the regions captured using MRI. For each animal, three snapshots were taken for each region from each section and results averaged. Image analyses were performed blinded using ImageJ (Fiji; ImageJ 1.53q) software.

### Tight junction protein

For tight junction protein quantification, the mean total percentage area of occludin, claudin-5 and ZO-1 was averaged across the three sections from the posterior cingulate/ temporal cortex and hippocampus brain regions of individual animals to assess any gross disruption to the BBB.

### Aquaporin-4

For AQP4 quantification, sections were co-stained for vasculature (using lectin) and AQP4 to allow perivascular AQP4 intensity (AQP4 surrounding blood vessels) to be quantified. Line profiles were manually drawn across five vessels in each lectin image using ImageJ (Fiji; ImageJ 1.53q). The line profiles were used to extract the intensity profile for both lectin and AQP4. Each profile was then normalised to the background intensity, defined as the average intensity at the edge of the profile, to account for differences in background intensity. Profiles were then centred around the maximum peak of lectin intensity profile which was defined as central reference point of each profile. For each animal, lectin and AQP4 profiles were averaged across five vessels per snapshot for three snapshots. The mean area under the curve (AUC) of the lectin and AQP4 profiles was calculated for each individual animal for each brain region.

### Microglia

To assess microglial activation due to lung infection and genotype, Iba1 positive cells were segmented and the cell perimeters calculated. Images were processed in ImageJ (Fiji; ImageJ 1.53q) using rolling ball background subtraction (radius 60 μm) before auto-thresholding with the Li method to extract ROIs for perimeter measures. Objects larger than 1500 pixels were excluded of the analysis as staining artefacts.

### *Streptococcus Pneumoniae* bacteria accumulation in lungs

Immunofluorescence was performed to quantify the extent of the *S. pneumoniae* bacteria accumulation the lungs of the rats during infection and reinfection, since the animals exhibited no overt behavioural changes during infection period. Rats (*n* = 3/group at 13-months and 18- months) were perfused with PBS before removing the lungs and drop fixing in 10% buffered formalin. The left lung was dissected then processed by the solution processor and embedded in paraffin wax. Sections (8 μm) of the lungs were collected using a rotary microtome and mounted on glass slides. Lung tissue sections were deparaffinised and rehydrated, then antigen retrieval was carried out by heating sections to 97.5 °C for 20 min in buffer (10 mM TRIS, 1 mM EDTA at pH 9). Following a PBS wash, slides were blocked with 200 μl primary antibody buffer (PBS + 1% BSA + 0.3% Triton) + 5% normal donkey serum, then incubated overnight in primary antibody (1:500 *Streptococcus pneumoniae)*. Slides were washed in PBS-T and incubated in secondary antibody (1:500 donkey anti-rabbit 488) and (1:500 donkey anti-rat 594 to stain antibodies against the bugs) in PBS + 0.3% Triton) for 2 h and allowed to dry at 37 °C overnight. Sections were then counterstained with DAPI before dehydration and cover-slipping.

To verify *S. pneumoniae* accumulation, stained sections were digitised using the fluorescence slide scanner (3D Histech) at 20x magnification. CaseViewer (version 2.4) was used to visualise *S. pneumoniae* colonies in each slice.

### Power calculation

Sample sizes were calculated in G*Power (v. 3.1.9.4). The primary endpoint was to detect an effect of infection on the BBB water exchange rate at 13-months timepoint as a main effect in an ANOVA analysis. Assuming within group standard deviation of 0.34 s^-1^ based on previous data^31^, a power of 0.8, significance threshold of 0.05 and effect size *f* = 0.43, a sample size of 11 per group was calculated.

### Statistical analysis

Two-way ANOVA was used to assess the main effects and interactions of genotype and infection on the imaging metrics (*K*^trans^, *v*_p_, *T*_1_, *k*_in_, *D*_e_, *D*_i_) and immunofluorescence markers (occludin, claudin-5, ZO-1, AQP4 AUC and Iba1+ perimeter). Separate ANOVAs were performed for each experimental timepoint and for each brain region for the immunofluorescent markers to assess each the impact of genotype and infection. Post-hoc Sidák’s multiple comparisons test was used when the main effect (infection or genotype) and/or interaction were significant. Pearson’s correlation coefficients were calculated between *K*^trans^ and *k*_in_ to test the association between the two BBB permeability metrics, and the association between *T*_1_ and *k*_in_ was determined to assess whether *k*_in_ measurements depend on *T*_1_.

Since AQP4 regulates water flux into the brain, evidence for correlation between the hippocampus AQP4 AUC and *k*_in_ was assessed to determine any association between non-invasive MRI measures and AQP4 protein expression. A Pearson's correlation was also used to assess the association between the hippocampal microglia (Iba1+) perimeter and AQP4 AUC. Finally, two-way ANOVA was used to assess the effect of aging in non-infected and infected conditions for *k*_in_, hippocampal AQP4 AUC and hippocampal Iba1+ perimeter (presented in Supplementary Materials, Supplementary Fig. S5). All parameters are reported as mean ± standard error of mean (S.E.M) and statistically significant results were noted when *P* < 0.05 throughout.

## Supplementary information

Supplementary Materials

## Data Availability

The datasets generated and analysed during the current study are available from the corresponding author, upon reasonable request.

## Code availability

The underlying code for the FEXI analysis in this study may be made available on reasonable request from the corresponding author.
