# Supplementary material for: MRI detects blood-brain barrier alterations in a rat model of Alzheimer’s disease and lung infection
Source: Npj Imaging. 2025 Mar 4;3:8. doi: 10.1038/s44303-025-00071-5 (PMC11879872; doi:10.1038/s44303-025-00071-5)
Supplement: Supplementary file 1 — Supplementary Materials [file 44303_2025_71_MOESM1_ESM.pdf]

Supplementary Materials

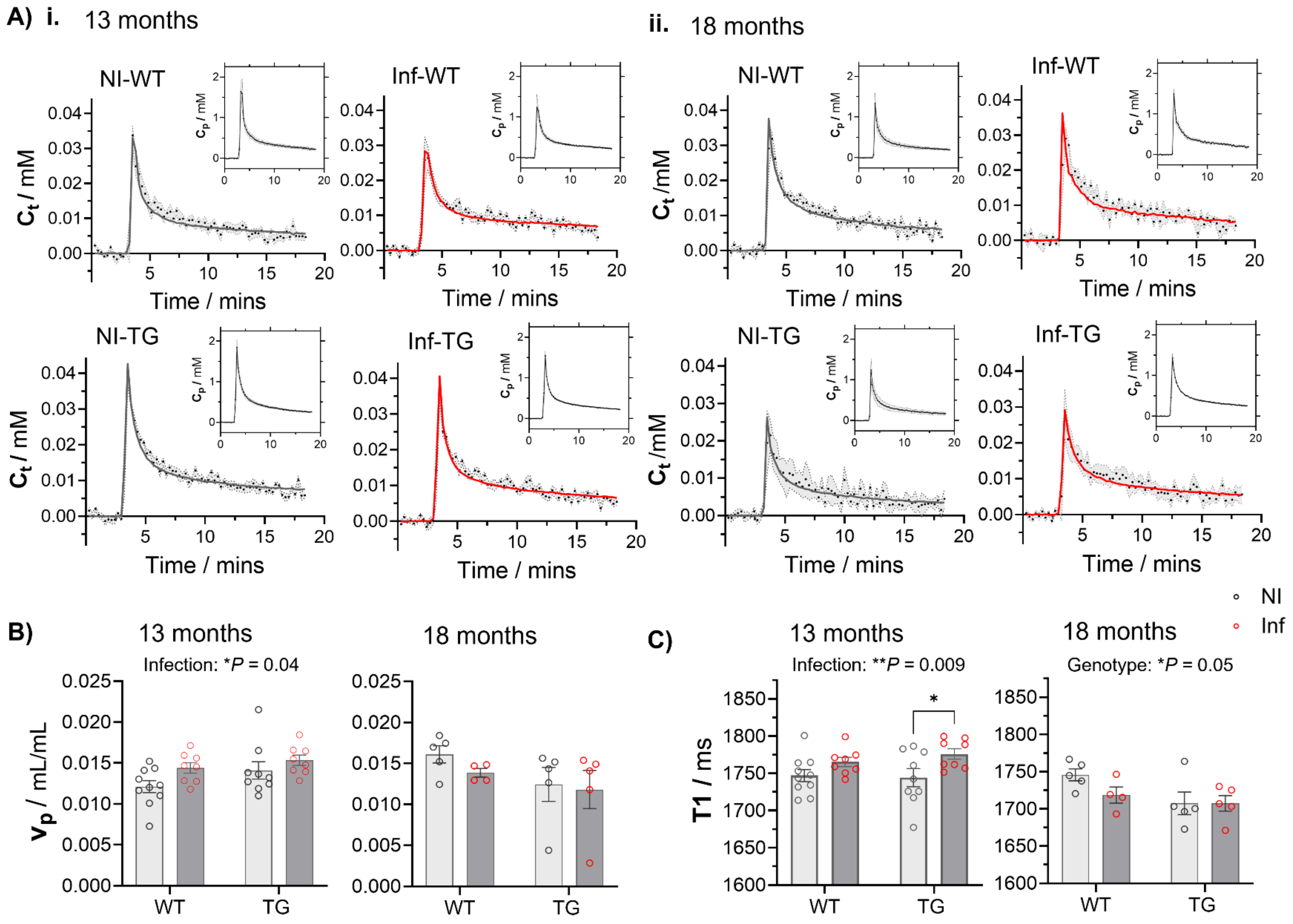

Supplementary Figure S1: DCE-MRI parameters (A) Mean dynamic time course of the tissue concentration (C_t) for non-infected wildtype (NI-WT), infected wildtype (Inf-WT), non-infected TgF344-AD (NI-TG) and infected TgF344-AD (Inf-TG) rats at **i.** 13-months and **ii.** 18 months. Plots show mean values at each dynamic timepoint (black dot) with associated error, S.E.M (grey shaded area). Inlays show mean arterial input function (AIF) for each group with plasma concentration (C_p) at each timepoint (mins) (**B)** Blood plasma volume (v_p) for non-infected (NI, black circle) and infected (Inf, red circle), wildtype (WT) and TgF344-AD (TG) rats at 13-months and 18-months old, at the 13-months infection led to significant increase (P = 0.04; ANOVA), but at 18-months timepoints differences were no longer present. (**C)** T_1 relaxation in the non-infected (NI, black circle) and infected (Inf, red circle), WT and TG animals, at 13-months infection led to significant increase (P = 0.009; ANOVA with *adjusted P = 0.04; post-hoc) and 18-months timepoints transgenic animals had significantly lower T1. Individual animal values are presented with mean value ± S.E.M, and significant ANOVA results indicated on each plot.

Supplementary Table S1: Quantification of blood plasma volume (v_p) and tissue longitudinal relaxation (T1) and statistical analysis.

Imaging Parameter		Values (Mean ± s.e.m)				Two-way ANOVA				
		Wild-type		TgF344-AD					Multiple comparisons	
		Non-infected	Infected	Non-infected	Infected		F-value	P-value		Adjusted P-value
v_p (%)	13 months	(n = 10) 1.21 ± 0.07	(n = 8) 1.44 ± 0.06	(n = 9) 1.41 ± 0.01	(n = 8) 1.53 ± 0.06	Infection	4.82	*0.04	Non-infected – infected	
						Genotype	3.23	0.08	WT	0.10
						Interaction	0.43	0.52	TG	0.50
	18 months	(n = 5) 1.61 ± 0.01	(n = 4) 1.39 ± 0.01	(n = 5) 1.24 ± 0.02	(n = 5) 1.18 ± 0.02	Infection	0.21	0.42	-	
						Genotype	2.63	0.13		
						Interaction	0.21	0.65		
T1 (ms)	13 months	(n = 10) 1747 ± 8	(n = 8) 1765 ± 6	(n = 9) 1744 ± 12	(n = 8) 1776 ± 7	Infection	7.80	** 0.009	Non-infected – infected	
						Genotype	0.20	0.66	WT	0.29
						Interaction	0.58	0.58	TG	*0.04
	18 months	(n = 5) 1746 ± 8	(n = 4) 1719 ± 11	(n = 5) 1707 ± 15	(n = 5) 1707 ± 10	Infection	1.36	0.81	WT - TG	
						Genotype	4.81	*0.05	Non-infected	0.06
						Interaction	1.36	0.26	Infected	0.76

Abbreviations: wild-type (WT) and transgenic TgF344-AD (TG)

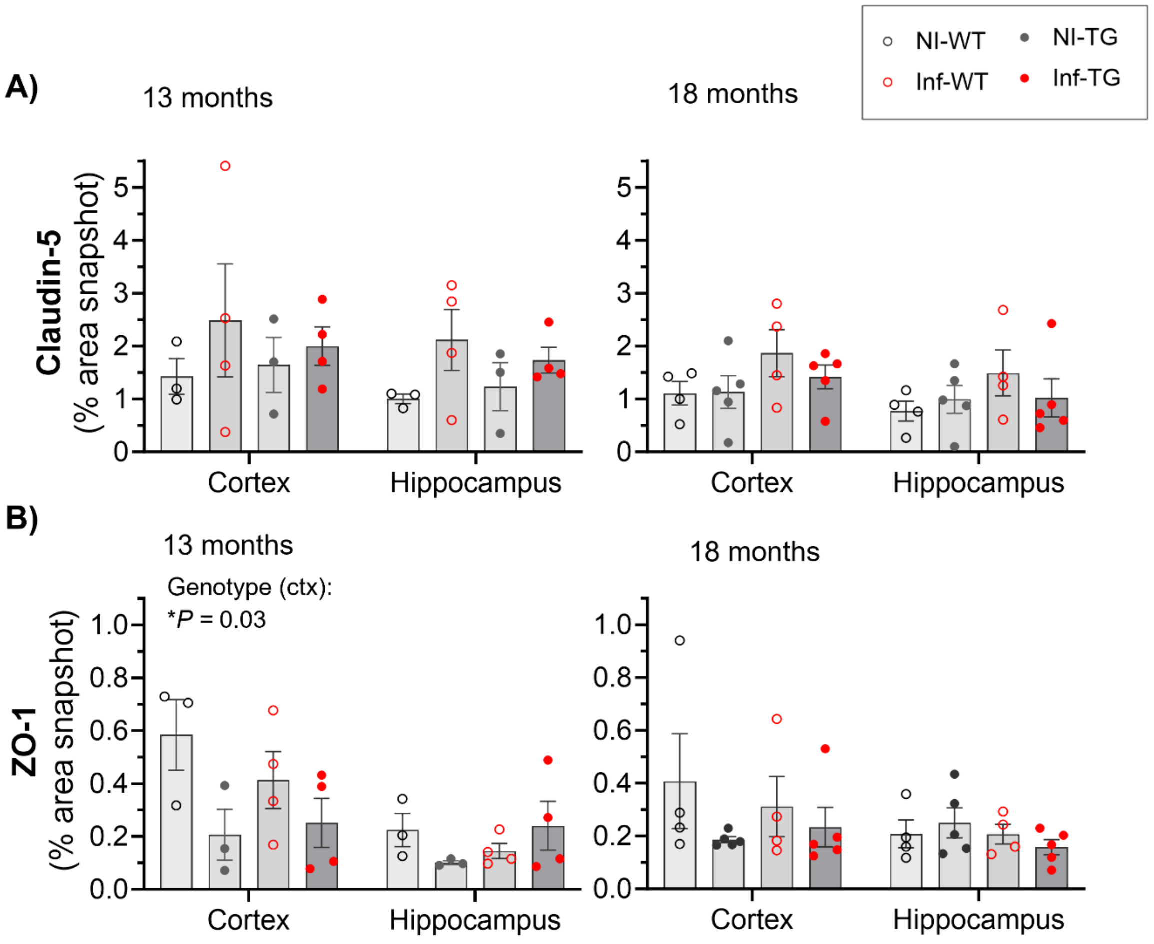

Supplementary Figure S2: Tight junction proteins across brain regions (A) Claudin-5 and **(B)** Zona-occluden-1 (ZO-1) measured in non-infected WT (NI-WT), non-infected TG (NI-TG), infected WT (Inf-WT) and infected TG group at 13-month and 18-month timepoints. 2-way ANOVA found a significant decrease ZO-1 between the WT and TG groups at 13-months in the cortex (ctx). All plots indicate individual animal values and mean ± S.E.M.

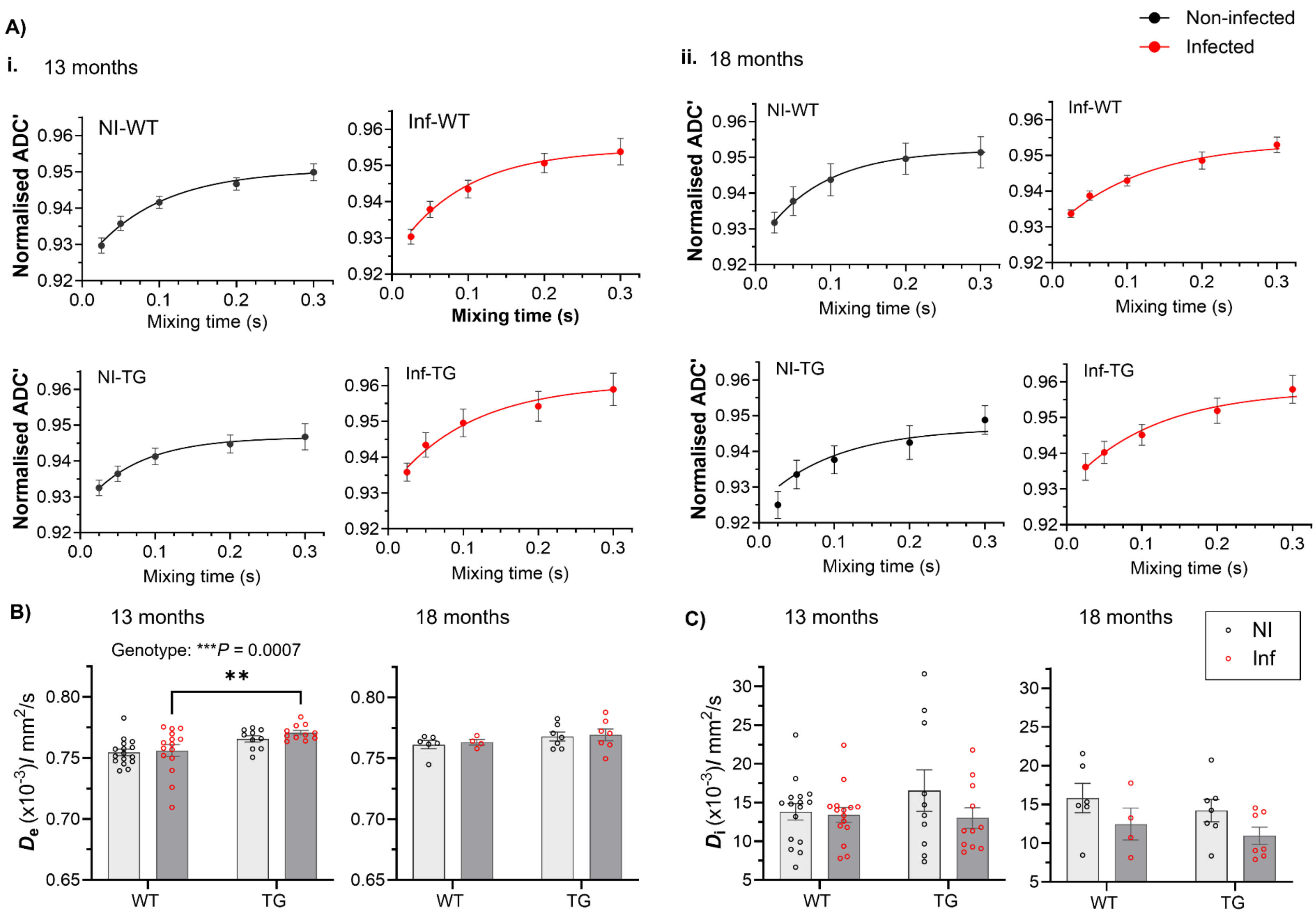
A)
Non-infected
Infected
i. 13 months
ii. 18 months
NI-WT
Inf-WT
NI-TG
Inf-TG
Normalised ADC'
Mixing time (s)
B)
13 months
18 months
Genotype: ***P = 0.0007
**
De (x10-3)/ mm2/s
WT
TG
C)
13 months
18 months
NI
Inf
Di (x10-3)/ mm2/s

Supplementary Figure S3: FEXI for BBB water exchange rate model. A) Normalised apparent diffusion coefficient (ADC') against mixing time for non-infected wild-type (NI-WT), infected wild-type (Inf-WT) and transgenic TgF344-AD (TG) in infected and non-infected conditions at **i.** 13-months and **ii.** 18-months old. **B)** Individual extravascular diffusivities (D_e) for wild-type (WT) and transgenic TgF344-AD (TG) in non-infected (NI, black circle) and infected (Inf, red circle) conditions, at 13 months there is a significant genotype effect ($P = 0.0007$; ANOVA with **adjusted $P = 0.0098$; post hoc multiple comparisons), at 18 months there were no difference due to either infection or genotype. **C)** Individual intravascular diffusivity (D_i) for non-infected (black circle) and infected (red circle) conditions, there were no difference due to either infection or genotype at either timepoints. Plots show mean ± S.E.M.

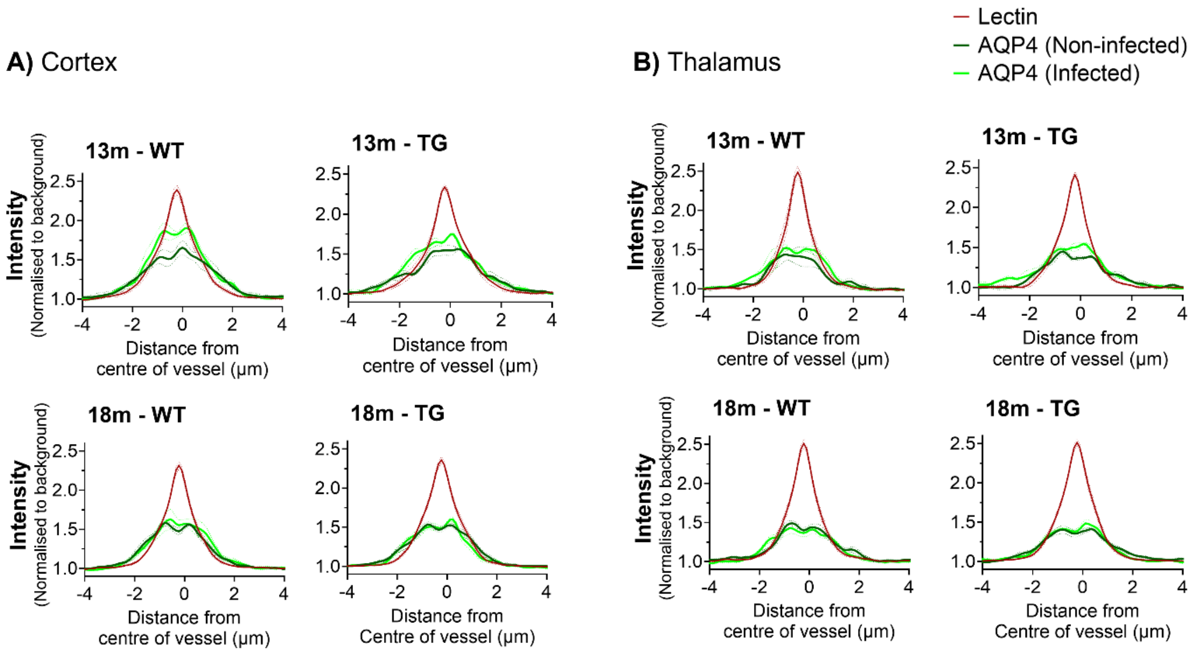

Supplementary Figure S4: Aquaporin-4 (AQP4) water channel protein profiles. Mean intensity profiles in non-infected and infected wildtype (WT) and transgenic TgF344-AD (TG) animals with vessel profile lectin (red) and AQP4 (dark green - non-infected; light green – infected) in **A)** cortex and **B)** thalamus brain regions.

Supplementary Table S2: Quantification of aquaporin-4 water channels and statistical analysis.

Aquaporin-4 A.U.C		Values (Mean ± s.e.m)				Two-way ANOVA				
		Wild-type		TgF344-AD					Multiple comparisons	
		Non-Infected	Infected	Non-infected	Infected		F-value	P-value		Adjusted P-value
**13 months**	Cortex									
		(*n* = 3)	(*n* = 3)	(*n* = 3)	(*n* = 3)	Infection	4.78	0.06	-	
		17.8 ± 1.4	21.8 ± 2.7	15.7 ± 1.1	18.7 ± 0.6	Genotype	2.52	0.15		
						Interaction	0.10	0.76		
	Hippocampus									
		(*n* = 3)	(*n* = 3)	(*n* = 3)	(*n* = 3)	Infection	14.0	**0.006	Non-infected – infected	
		7.8 ± 1.3	10.7 ± 2.0	6.0 ± 0.3	12.3 ± 0.5	Genotype	0.006	0.94	WT	0.24
						Interaction	1.83	0.21	TG	*0.01
	Thalamus									
		(*n* = 3)	(*n* = 3)	(*n* = 3)	(*n* = 3)	Infection	6.94	*0.03	Non-infected – infected	
		8.5 ± 1.9	11.8 ± 1.2	9.7 ± 0.2	12.9 ± 1.1	Genotype	0.92	0.36	WT	0.19
						Interaction	0.0004	0.98	TG	0.19
**18 months**	Cortex									
		(*n* = 6)	(*n* = 7)	(*n* = 4)	(*n* = 5)	Infection	0.002	0.96	-	
		13.2 ± 0.9	13.9 ± 2.3	13.1 ± 0.7	12.3 ± 0.5	Genotype	0.62	0.44		
						Interaction	0.54	0.47		
	Hippocampus									
		(*n* = 6)	(*n* = 7)	(*n* = 4)	(*n* = 5)	Infection	7.09	*0.02	Wildtype - transgenic	
		7.7 ± 0.5	9.1 ± 0.8	5.6 ± 0.4	7.7 ± 1.0	Genotype	7.36	*0.01	NI	*0.04
						Interaction	0.37	0.37	Inf	0.34
	Thalamus									
		(*n* = 6)	(*n* = 7)	(*n* = 4)	(*n* = 5)	Infection	0.40	0.54	-	
		11.1 ± 1.5	9.5 ± 1.1	10.9 ± 0.8	11.1 ± 0.8	Genotype	0.42	0.53		
						Interaction	0.62	0.44		

Abbreviations: wild-type (WT) and transgenic TgF344-AD (TG)

Supplementary Table S3: Quantification of microglia (Iba stain) perimeter and statistical analysis.

Iba perimeter	Region	Wild-type Non-Infected	Wild-type Infected	TgF344-AD Non-infected	TgF344-AD Infected	Two-way ANOVA	F-value	P-value	Multiple comparisons	Adjusted P-value
13 months	Cortex	(*n* = 3) 227 ± 3	(*n* = 3) 172 ± 3	(*n* = 3) 193 ± 6	(*n* = 3) 200 ± 16	Infection	7.0	*0.03	Non-infected – infected	
						Genotype	0.1	0.74	WT	*0.005
						Interaction	11.6	**0.009	TG	0.85
	Hippocampus	(*n* = 3) 225 ± 5	(*n* = 3) 163 ± 6	(*n* = 3) 183 ± 7	(*n* = 3) 195 ± 19	Infection	5.5	*0.05	Non-infected – infected	
						Genotype	0.2	0.65	WT	**0.007
						Interaction	11.5	**0.009	TG	0.73
	Thalamus	(*n* = 3) 203 ± 7	(*n* = 3) 143 ± 7	(*n* = 3) 163 ± 4	(*n* = 3) 163 ± 11	Infection	14.2	**0.006	Non-infected – infected	
						Genotype	1.6	0.23	WT	**0.001
						Interaction	14.5	**0.005	TG	0.99
18 months	Cortex	(*n* = 6) 211 ± 9	(*n* = 7) 177 ± 9	(*n* = 4) 176 ± 10	(*n* = 5) 180 ± 11	Infection	2.3	0.15	-	
						Genotype	2.5	0.13		
						Interaction	3.5	0.08		
	Hippocampus	(*n* = 6) 198 ± 11	(*n* = 7) 190 ± 15	(*n* = 4) 171 ± 11	(*n* = 5) 171 ± 11	Infection	0.1	0.75	-	
						Genotype	3.5	0.07		
						Interaction	0.1	0.75		
	Thalamus	(*n* = 6) 166 ± 6	(*n* = 7) 158 ± 1.1	(*n* = 4) 155 ± 13	(*n* = 5) 150 ± 13	Infection	0.3	0.57	-	
						Transgene	0.6	0.43		
						Interaction	0.01	0.90		

Abbreviations: wild-type (WT) and transgenic TgF344-AD (TG)

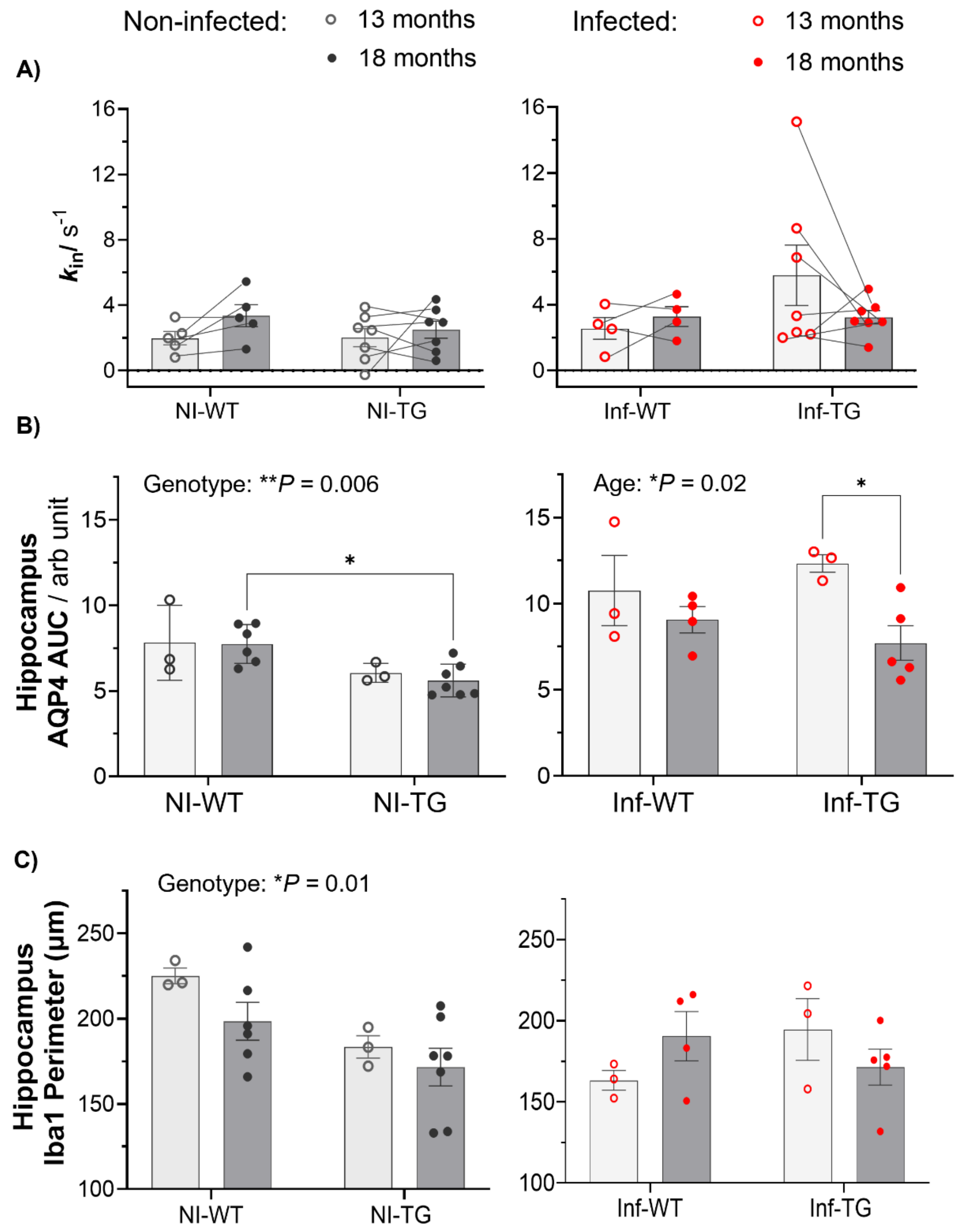

Supplementary Figure S5: Alterations to water exchange rate, aquaporin-4 water channel protein and microglia when animals age. (A) Paired measurements of BBB water exchange rate (k_{in}) at 13-months and 18-months old for non-infected wildtype (NI-WT), non-infected TgF344-AD (NI-TG) and infected wildtype (Inf-WT) and infected TgF344-AD (Inf-TG), no significant differences in k_{in} were found due to age or genotype. **(B)** Hippocampus aquaporin-4 (AQP4) AUC was significantly lower due to genotype in non-infected group (adjusted *P = 0.02) and significantly lower due to age in infected group (adjusted *P = 0.03) **(C)** Mean hippocampus microglia measured by Iba1+ cells perimeter. Perimeter significantly lower in TgF344-AD animals due genotype in the non-infected group. Individual animal values are presented with mean value ± S.E.M, and significant ANOVA results indicated on each plot.

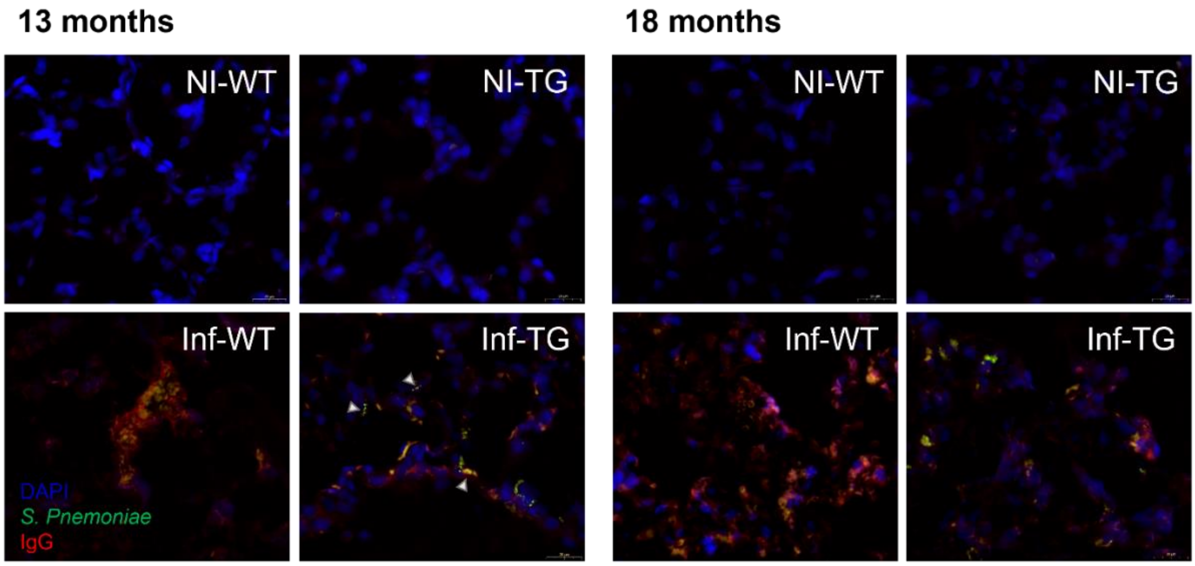

Supplementary Figure S6: Streptococcus pneumonia bacteria accumulation in lung tissue. Representative example fluorescence immunohistochemistry staining of the lung tissue for non-infected wildtype (NI-WT), non-infected TgF344-AD (NI-TG), infected wildtype (Inf-WT) and infected TgF344-AD (Inf-TG) rats with DAPI staining for cell nuclei (blue), *Streptococcus pneumoniae* staining for bacteria (green) and IgG antibodies staining (red). White arrows indicate some of the locations the chains of bacteria and the regions of both bacteria and antibodies (yellow).

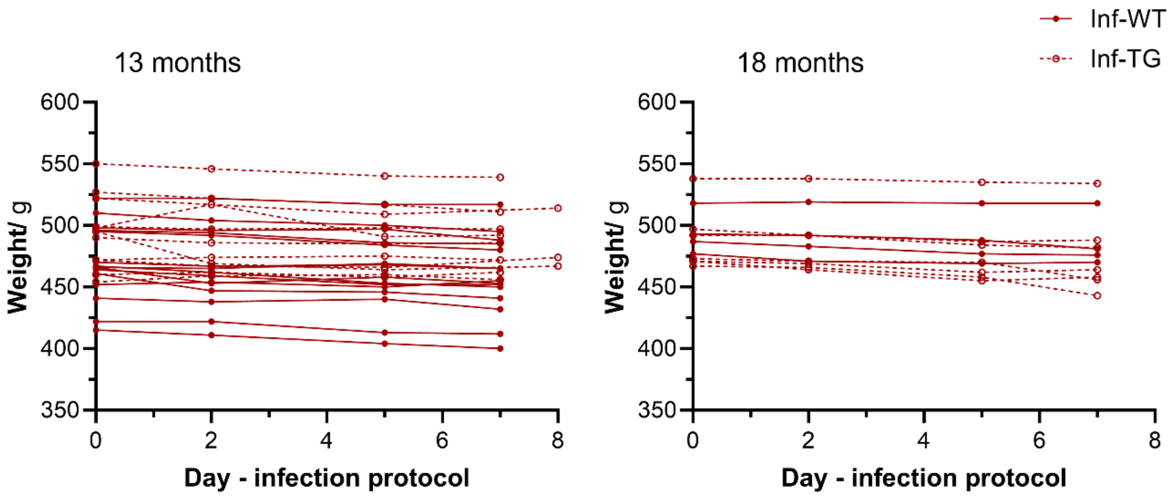

Supplementary Figure S7: Monitoring weights of individual infected wildtype (Inf-WT) and infected TgF344-AD (Inf-TG) animal during infection protocol, from baseline (day 0) until imaging (day 7/8).

Supplementary Table S4: List of antibodies used for the immunohistochemistry
**Streptococcus pneumoniae* types 3, 4, 6, 7, 9, 14, 18 and 23 surface antigens.

<table>
<tr><th></th><th colspan="3">Primary antibodies</th><th colspan="2">Secondary antibodies</th></tr>
<tr><th>Target protein</th><th>Species raised in</th><th>Dilution</th><th>Manufacturer (Reference)</th><th>Manufacturer & Reference</th><th>Dilution</th></tr>
<tr><td>Lectin</td><td>tomato</td><td>1:250</td><td>ThermoFisher-Invitrogen (L32478)</td><td>-</td><td>-</td></tr>
<tr><td>Occludin</td><td>rabbit</td><td>1:500</td><td>ThermoFisher-Invitrogen (71-1500)</td><td rowspan="6">Molecular Probes - Invitrogen

Goat anti-rabbit (SAB4600107)

goat anti-mouse 594 (SAB4600105)</td><td rowspan="6">1:500</td></tr>
<tr><td>Claudin-5</td><td>mouse</td><td>1:500</td><td>ThermoFisher-Invitrogen (71-1500)</td></tr>
<tr><td>ZO-1</td><td>mouse</td><td>1:1000</td><td>Thermofisher, (339100 (WA316684))</td></tr>
<tr><td>Aquaporin-4</td><td>rabbit</td><td>1:1000</td><td>Millipore (AB3594)</td></tr>
<tr><td>Iba1+</td><td>rabbit</td><td>1:500</td><td>Abcam (ab178846)</td></tr>
<tr><td>*Strep. pneumoniae</td><td>rabbit</td><td>1:500</td><td>(A21206)</td><td>Donkey anti-rabbit</td><td>1:500</td></tr>
<tr><td>IgG</td><td>donkey</td><td>1:500</td><td>Abcam (ab20429)</td><td>Donkey anti-rat</td><td>1:500</td></tr>
</table>
